# Targeting the Inflammation–Metabolism Axis in MGUS: Causal Roles of CXCL10 Mediated by Blood Metabolites

**DOI:** 10.1155/mi/8804923

**Published:** 2025-12-08

**Authors:** Siyu Gu, Zhongyuan Wang, Zhiyao Wen, Mingyuan Fang, Long Ye, Jie Jiang, Haiying Hua

**Affiliations:** ^1^ Department of Hematology, Affiliated Hospital of Jiangnan University, Wuxi, Jiangsu, China, jiangnan.edu.cn; ^2^ Wuxi School of Medicine, Jiangnan University, Wuxi, Jiangsu, China, jiangnan.edu.cn

**Keywords:** CXCL10, IL-6, inflammatory cytokines, Mendelian randomization, metabolic mediators, MGUS

## Abstract

**Background:**

Inflammatory cytokines have been implicated in monoclonal gammopathy of undetermined significance (MGUS), but their causal mechanisms remain unclear. Metabolites play pivotal roles in plasma cell dysregulation, however, their potential mediation effects between cytokines and MGUS are unexplored. We aimed to elucidate causal relationships between inflammatory cytokines and MGUS and identify metabolite‐mediated pathways.

**Methods:**

Using genome‐wide association study (GWAS) summary statistics, we performed bidirectional two‐sample Mendelian randomization (MR) to assess causality between 91 inflammatory cytokines and MGUS. A two‐step MR approach was employed to investigate metabolite mediation using data from 1400 blood metabolites. Sensitivity analyses addressed pleiotropy and reverse causality (IVs: *p* < 1 × 10^−5^, *F*‐statistic > 10).

**Results:**

MR analysis identified CXCL10 (OR = 2.12, 95% CI: 1.06–4.23, *p* = 0.034) and IL‐6 (OR = 3.61, 95% CI: 1.22–10.65, *p* = 0.020) as causal risk factors for MGUS. We also found Threonate (OR = 2.24, 95% CI: 1.06–4.75, *p* = 0.035), X‐22776 (OR = 3.45, 95% CI: 1.37–8.67, *p* = 0.009) and glucose to sucrose ratio (OR = 2.89, 95% CI: 1.18–7.07, *p* = 0.020) were associated with increased MGUS risk, while N‐acetylputrescine to (N(1) + N(8))‐acetylspermidine ratio (OR = 0.65, 95% CI: 0.43–0.98, *p* = 0.039) showed protective effects. Mediation analysis revealed 2 metabolites Threonate and X‐22776 mediating CXCL10’s effect on MGUS. Threonate mediated 11.2% (*β* = 0.08, *p* = 0.014) and X‐22776 mediated 17.7% (*β* = 0.13, *p* = 0.028) of CXCL10’s total effect. Sensitivity analyses confirmed robustness (no pleiotropy: MR‐Egger intercept *p* > 0.05; Cochran’s *Q* *p* > 0.05).

**Conclusion:**

This study deeply reveals the mechanism by which inflammatory cytokines affect the pathogenesis of MGUS through metabolite‐mediated pathways, providing new potential targets for the early diagnosis and treatment of MGUS. In the future, other inflammatory cytokines and metabolites that may be related to the pathogenesis of MGUS can be further explored, and the interactions and potential mechanisms between them can be further studied to provide a more comprehensive theoretical basis and practical guidance for the prevention and treatment of MGUS.

## 1. Introduction

Monoclonal gammopathy of undetermined significance (MGUS) is a premalignant condition characterized by the clonal proliferation of plasma cells or lymphoplasmacytic cells in the bone marrow, marked by the abnormal production of monoclonal immunoglobulin without evidence of end‐organ damage such as hypercalcemia, renal insufficiency, anemia, or bone lesions [1]. Classified into IgM and non‐IgM subtypes based on monoclonal protein type, IgM‐MGUS is often linked to lymphoproliferative disorders, while non‐IgM‐MGUS may progress to multiple myeloma (MM) [2]. Although most patients remain asymptomatic, ~1% of individuals over 50 years old develop MGUS, with a 1% annual risk of progression to MM or related malignancies, a process closely related to genetic variations, immune microenvironment disturbances, and the activation of inflammatory factors [3, 4]. Despite its clinical significance, the molecular mechanisms driving the transition from MGUS to overt malignancy are unclear, highlighting the need to identify early biomarkers and modifiable risk factors.

Recent proteomics studies have found that the transition from MGUS to MM involves the upregulation of proteins related to the EIF2 signaling pathway, DNA repair, and translation quality control, suggesting that proteostasis imbalance may play a key role in disease transformation [5]. Emerging evidence suggests that chronic inflammation plays a pivotal role in the pathogenesis of plasma cell dyscrasias. Elevated levels of pro‐inflammatory cytokines, including interleukin‐6 (IL‐6) and tumor necrosis factor‐*α* (TNF‐*α*), have been observed in individuals with MGUS, potentially promoting plasma cell survival and monoclonal protein secretion through pathways such as NF‐κB and JAK‐STAT signaling [6, 7]. In parallel, metabolic dysregulation has been linked to MGUS, with studies reporting alterations in lipid metabolism, amino acid pathways, and oxidative stress markers in affected individuals [3, 8, 9].

While these associations provide valuable insights, the causal relationships between inflammatory cytokines, metabolites, and MGUS remain uncertain. Observational studies are limited by reverse causation and unmeasured confounders, complicating efforts to disentangle direct biological effects from secondary phenomena [10]. Mendelian randomization (MR), a method leveraging genetic variants as instrumental variables (IVs) to infer causality, offers a robust approach to address these limitations [11]. Recent advances in genome‐wide association studies (GWAS) have enabled large‐scale analyses of inflammatory cytokines and metabolites, providing genetic instruments to explore their roles in disease pathogenesis [12, 13].

This study aims to elucidate the causal effects of 91 inflammatory cytokines on MGUS risk and investigate the mediating role of metabolites in these relationships. By integrating genetic data from GWAS of cytokines, metabolites, and MGUS, we seek to identify novel biomarkers and pathways underlying MGUS development, offering potential targets for early intervention and therapeutic strategies.

## 2. Methods

### 2.1. Study Design

**Figure 1:**
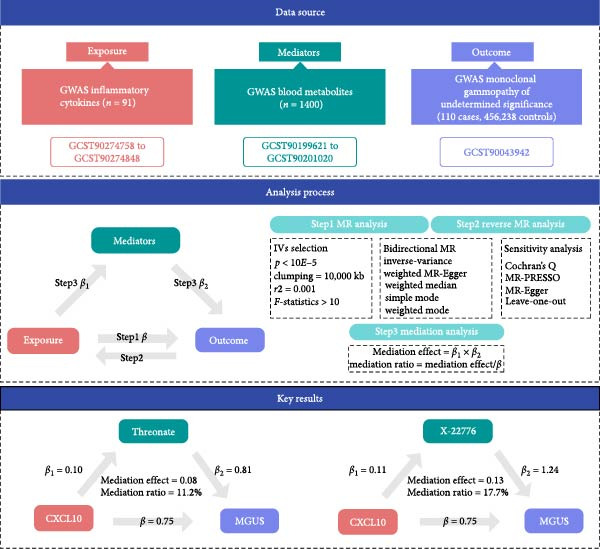
The data sources for the exposure, mediators, and outcome used in the present study. The processes of Mendelian randomization and mediation analysis. The key results of the causal pathways of Threonate and X‐22776 mediating CXCL10’s effect on MGUS.

To investigate causal relationships and mediation pathways, we implemented a two‐phase MR framework. In Phase 1, bidirectional two‐sample MR analyses were performed to evaluate the causal effects of 91 inflammatory cytokines on MGUS risk, using genetic instruments derived from GWAS. Phase 2 employed a two‐step MR approach to quantify metabolite‐mediated pathways linking cytokines to MGUS. Reverse MR analyses were conducted to exclude reverse causality, and mediation effects were computed by integrating estimates from cytokine‐to‐metabolite and metabolite‐to‐MGUS associations [14, 15] (Figure 1).

### 2.2. Data Sources

This study analyzed aggregated GWAS summary statistics from large‐scale datasets. All participants provided informed consent and the studies adhered to ethical standards without requiring additional approval. These datasets were accessed through the GWAS Catalog (https://www.ebi.ac.uk/gwas/).

Summary‐level GWAS data for inflammatory cytokines (Accession Codes: GCST90274758 to GCST90274848, PMID: 37563310) were obtained from an analysis of 14,824 European participants, identifying protein quantitative trait loci (pQTLs) for 91 circulating cytokines [16]. The original study recruited 11 cohorts with genome‐wide genetic data and plasma proteomic data. All participants provided written, informed consent, and each cohort was approved by specific ethics committees, which can be accessed in the supplementary file of Zhao et al. [16].

MGUS GWAS data (accession codes: GCST90043942, PMID: 31768069) were sourced from the analyze of 110 European ancestry cases and 456,238 European ancestry controls [17]. This study was approved by the University of Queensland Human Research Ethics Committee B (2011001173) and the Ethics Committee of Westlake University (20200722YJ001).

Metabolite GWAS summary statistics (Accession Codes: GCST90199621 to GCST90201020, PMID: 36635386) were based on data from the Canadian Longitudinal Study on Aging (CLSA), including measurements for 1091 blood metabolites and 309 metabolite ratios in 8299 individuals [13]. Plasma metabolite profiling was conducted using the ultrahigh performance liquid chromatography‐tandem mass spectroscopy (UPLC‐MS/MS) platform (Metabolon HD4) by Metabolon, Inc. (Durham, NC, USA). Strict quality control and curation procedures were applied to ensure accurate compound identification and to remove systemic artifacts and misassignments. Metabolite levels were batch‐normalized, log‐transformed, and standardized for subsequent genome‐wide association analyses. This study was approved by the research ethics boards of the Jewish General Hospital (2021–2762).

### 2.3. IV Selection

To optimize the identification of genetic instruments, we applied a genome‐wide suggestive threshold (*p* < 1 × 10^−5^) for selecting SNPs associated with the 91 inflammatory cytokines and 1400 metabolites, ensuring a comprehensive capture of genetic variation [18]. Candidate SNPs were subsequently subjected to rigorous quality control. Linkage disequilibrium (LD), reflecting the nonrandom co‐inheritance of nearby genetic variants, was addressed by clumping SNPs using the European 1000 Genomes reference panel (clumping = 10,000 kb, r2 = 0.001). Instrument strength was validated via *F*‐statistics (*F* > 10), calculated as *F* = *R* ^2^×(*N*−2)/(1−*R* ^2^), where *R* ^2^ (variance explained) was derived from 2 × MAF × (1 − MAF) × *β*2^2^, with *β* representing the SNP’s effect size [19]. The analysis checked for overlapping SNPs at each step from exposure to mediator and from mediator to outcome and removed any overlaps before analysis to ensure the results are robust.

### 2.4. Statistical Analysis

All MR analyses were conducted using R software (version 4.4.2) with the “*TwoSampleMR*” package. Bidirectional MR was first performed to assess causal relationships between inflammatory cytokines and MGUS. Causal estimates were derived using five complementary methods: inverse‐variance weighted (IVW, primary method), MR‐Egger, weighted median, simple mode, and weighted mode. The IVW approach assumes balanced pleiotropy and synthesizes effect estimates across all IVs through variance‐weighted averaging, providing maximum statistical power under valid IV assumptions. Heterogeneity across IVs was evaluated using Cochran’s Q statistic (*p* < 0.05 indicating significant heterogeneity). To address horizontal pleiotropy—where genetic variants influence outcomes through pathways independent of the exposure—we applied the MR‐PRESSO global test, which identifies outliers by comparing observed versus expected residual sums of squares under the null hypothesis of no pleiotropy. Additionally, the MR‐Egger intercept test was employed to detect directional pleiotropy, a nonzero intercept (*p* < 0.05) suggests systematic bias in causal estimates. Sensitivity of results to individual SNPs was assessed via leave‐one‐out analysis, iteratively excluding each variant to evaluate its influence on overall estimates. Finally, scatterplots and funnel plots were generated to visualize effect size distributions and pleiotropy patterns. Finally, mediation effects were calculated as the product of coefficients (*β* cytokine→metabolite × *β* metabolite→MGUS), with 95% confidence intervals generated via the delta method. The direct effect was defined as (total effect−mediation effect), and the proportion mediated was defined as (mediation effect/total effect) × 100% [20].

## 3. Results

### 3.1. Causal Effects of Inflammatory Cytokines on MGUS

A total of 1818 SNPs were selected from 91 inflammatory cytokines, following the exclusion of repetitive, ambiguous, and unproxied SNPs at a *p*‐value threshold of 1 × 10^−5^. All IVs exhibited strong associations (F‐statistic > 10; Supporting Information 1: Table S1). Bidirectional two‐sample MR analyses identified two inflammatory cytokines, C‐X‐C motif chemokine 10 (CXCL10) and interleukin‐6 (IL‐6), with causal associations with MGUS. Using IVW as the primary method, genetically predicted CXCL10 (OR = 2.12, 95% CI: 1.06–4.23, *p* = 0.034) and IL‐6 (OR = 3.61, 95% CI: 1.22–10.65, *p* = 0.020) were significantly associated with increased MGUS risk. Sensitivity analyses revealed no significant heterogeneity (Cochran’s *Q* *p* > 0.05) or horizontal pleiotropy (MR‐Egger intercept *p* > 0.05; MR‐PRESSO global test *p* > 0.05), and MR‐PRESSO detected no outliers (Supporting Information 2: Figure S1). Reverse MR analysis ruled out reverse causality between MGUS and CXCL10 (*p* > 0.05). However, a suggestive association was observed between MGUS and elevated IL‐6 levels. Scatter plots and funnel plots demonstrated consistent causal estimates across MR methods, and leave‐one‐out sensitivity analyses excluded disproportionate influence from individual SNPs (Supporting Information 3: Figures S2). Given the robust unidirectional causal relationship between CXCL10 and MGUS, subsequent analyses were focused on elucidating the metabolic pathways mediating CXCL10’s effect on MGUS pathogenesis.

### 3.2. Causal Effects of Metabolites on MGUS

MR analysis of 1400 blood metabolites (Supporting Information 4: Table S2) identified two metabolites and two metabolite ratios causally linked to MGUS. Genetically elevated levels of Threonate (OR = 2.24, 95% CI: 1.06–4.75, *p* = 0.035), X‐22776 (OR = 3.45, 95% CI: 1.37–8.67, *p* = 0.009, It is important to note that X‐22776 is an unnamed metabolite in the original dataset; its exact chemical structure and pathway remain uncharacterized.) and glucose to sucrose ratio (OR = 2.89, 95% CI: 1.18–7.07, *p* = 0.020) were associated with increased MGUS risk, while N‐acetylputrescine to (N(1) + N(8))‐acetylspermidine ratio (OR = 0.65, 95% CI: 0.43–0.98, *p* = 0.039) showed protective effects (Supporting Information 5: Figure S3). No heterogeneity or pleiotropy was detected (Cochran’s *Q* *p* > 0.05; MR‐Egger intercept *p* > 0.05; MR‐PRESSO global test *p* > 0.05).

### 3.3. Causal Effects of CXCL10 on Metabolites

Building on the identified causal relationship between CXCL10 and MGUS, we further investigated the mediating role of blood metabolites in this pathway. Using CXCL10 as the exposure and 1400 blood metabolites as outcomes, MR analyses revealed causal associations between CXCL10 and 2 metabolites and 2 metabolite ratios aforementioned. The IVW method demonstrated that genetically predicted CXCL10 levels were positively associated with Threonate (*β* = 0.10, 95% CI: 0.02–0.19, *p* = 0.014), X‐22776 (*β* = 0.11, 95% CI: 0.01–0.20, *p* = 0.028) and N‐acetylputrescine to (N(1) + N(8))‐acetylspermidine ratio (*β* = 0.08, 95% CI: 0.005–0.16, *p* = 0.038), while inversely correlated with glucose to sucrose ratio (*β* = −0.12, −0.20 to −0.04, *p* = 0.002). Sensitivity analyses confirmed minimal heterogeneity (Cochran’s *Q* *p* > 0.05) and no horizontal pleiotropy (MR‐Egger intercept *p* > 0.05; MR‐PRESSO global test *p* > 0.05) (Supporting Information 6: Figure S4).

### 3.4. Mediation Analysis

To elucidate the mechanisms underlying the causal effect of CXCL10 on MGUS, we conducted a two‐step MR mediation analysis. Using the product method, we identified two blood metabolites that mediated the causal pathway between CXCL10 and MGUS. Genetically predicted CXCL10 levels increased the risk of MGUS by elevating Threonate (*β* = 0.08, SE = 0.31, mediation ratio = 11.2%) and X‐22776 (*β* = 0.13, SE = 0.58, mediation ratio = 17.7%). Our findings highlight the dual role of CXCL10 in driving MGUS risk through metabolite‐mediated pathways, suggesting potential therapeutic targets for disrupting inflammation‐metabolism crosstalk in early plasma cell dyscrasias.

## 4. Discussion

Our study provides novel insights into the causal relationships between inflammatory cytokines, metabolites, and MGUS, revealing CXCL10 and IL‐6 as pivotal mediators in MGUS pathogenesis. As a precursor to MM and related plasma cell dyscrasias, MGUS represents a critical window for intercepting malignant progression through targeted interventions [21–23]. The inflammatory microenvironment, driven by cytokines like IL‐6 and CXCL10, appears to synergize with metabolic dysregulation in plasma cell transformation, potentially mediated through NF‐κB and JAK‐STAT signaling cascades [24–27].

The causal association between IL‐6 and MGUS risk aligns with its established role in plasma cell survival and monoclonal protein secretion [25, 26]. IL‐6 activates both JAK‐STAT and NF‐κB pathways, creating a feedforward loop that sustains chronic inflammation while simultaneously reprograming cellular metabolism [25, 27, 28]. This dual functionality may explain its prominence in our MR analysis, as IL‐6‐driven metabolic alterations in lipid and amino acid pathways could provide the energetic substrates required for premalignant plasma cell expansion [29–31]. Recent preclinical studies demonstrate that IL‐6/JAK‐STAT3 axis inhibition reduces myeloma cell viability while normalizing inflammation‐associated metabolic disturbances, supporting its therapeutic potential in precursor states [25, 32].

CXCL10’s strong association with MGUS risk highlights the underappreciated role of chemokine‐driven inflammation in plasma cell dyscrasias. As an IFN‐γ‐inducible chemokine, CXCL10 recruits immune cells that secrete additional pro‐inflammatory mediators while directly activating NF‐κB in plasma cells [28, 33]. This creates a self‐perpetuating inflammatory niche that may drive genomic instability and clonal evolution [24, 34]. Notably, our mediation analysis suggests CXCL10’s effects are partially mediated through metabolites like threonate and glucose/sucrose ratios, implicating oxidative stress and energy metabolism as key amplifiers of chemokine signaling [29, 31, 35].

The metabolic perturbations identified—particularly in lipid metabolism and amino acid pathways—corroborate emerging evidence of inflammation‐metabolism crosstalk in plasma cell disorders [29–31]. NF‐κB activation by inflammatory cytokines directly upregulates lipid synthesis enzymes like ACC1, while JAK‐STAT signaling alters amino acid transporter expression, creating metabolic dependencies that could be therapeutically targeted [28, 31, 36]. Our study identified specific metabolites that mediate the causal relationship between CXCL10 and MGUS. Elevated levels of Threonate and X‐22776, as well as the glucose to sucrose ratio, were found to be associated with an increased risk of MGUS. X‐22776 is a mediator of CXCL10’s effect, although its exact chemical identity remains unknown. This limitation reflects a common challenge in untargeted metabolomics studies where a significant proportion of detected features await structural characterization. Threonate, as a primary metabolite of vitamin C, is intricately involved in antioxidant defense [37]. Magnesium‐L‐threonate is a compound of L‐threonic acid and magnesium, may indirectly influence systemic inflammation and metabolic pathways associated with neurodegenerative diseases [38]. Furthermore, the impact of Magnesium‐L‐threonate on key metabolic pathways such as glutathione metabolism and the tricarboxylic acid cycle aligns with the reported metabolic reprograming in MGUS‐to‐MM progression, suggesting a plausible role in suppressing pro‐tumor metabolic adaptation [39]. Conversely, the N‐acetylputrescine to (N(1) + N(8))‐acetylspermidine ratio showed protective effects suggests polyamine metabolism modulation might counteract inflammation‐driven transformation, a hypothesis supported by recent studies showing polyamine blockade inhibits myeloma cell proliferation [22, 31].

These mechanistic insights have direct clinical implications. Current risk stratification models for MGUS/Smoldering Multiple Myeloma (SMM) increasingly incorporate inflammatory and metabolic biomarkers, with our findings supporting CXCL10 and IL‐6 as potential candidates for dynamic monitoring [22, 23, 34]. The effect sizes found in this study (CXCL10: OR = 2.12; IL‐6: OR = 3.61) are considered clinically meaningful in epidemiological studies and are comparable to the effect sizes of some established cancer risk factors [40], suggesting that measuring plasma CXCL10 and IL‐6 levels may help improve existing risk stratification models for MGUS progression. The favorable toxicity profile of immunomodulatory drugs (IMiDs) like lenalidomide makes them rational candidates for early intervention, particularly given their dual anti‐inflammatory and metabolic effects [41–43]. Phase II trials demonstrating lenalidomide’s efficacy in high‐risk SMM align with our proposed inflammation‐metabolism axis targeting, as (IMiDs) simultaneously degrade IKZF1/3 transcription factors and modulate cereblon‐mediated metabolic adaptations [41, 42]. IL‐6 receptor antagonists (such as tocilizumab) are well‐established anti‐inflammatory drugs, and their repurposing for the prophylactic treatment of high‐risk MGUS patients warrants exploration [44]. Although specific inhibitors for CXCL10 are not yet mature, its receptor CXCR3 is a validated drug target [45]. Therefore, our study not only identifies novel risk factors but also provides potential translational directions for risk stratification and targeted intervention in the precancerous stage of MGUS.

While our study offers valuable insights into the molecular mechanisms underlying MGUS, it is essential to recognize certain limitations. First, the MR approach is based on the assumption that genetic variants serve as valid IVs, which may be influenced by pleiotropy or population stratification. Second, the GWAS summary data for MGUS lacks critical clinical metadata, such as subtype stratification (e.g., IgM vs. non‐IgM), age distribution, and follow‐up duration, which limits our ability to evaluate the representativeness of cases or investigate subtype‐specific causal mechanisms. Finally, the identified metabolites and their specific roles in MGUS pathogenesis necessitate further validation in experimental models and clinical studies.

In conclusion, our work establishes CXCL10 and IL‐6 as central drivers of MGUS pathogenesis through intertwined inflammatory and metabolic mechanisms. Targeting this inflammation‐metabolism axis via cytokine inhibition, metabolic reprograming, or IMiD‐based therapies could disrupt the MGUS‐MM continuum, offering new strategies for preventing plasma cell malignancy progression.

## Ethics Statement

All data of present study used publicly available data with preexisting ethical approvals. No additional ethical approval was necessary for this analysis.

## Consent

As the study employed existing GWAS data with proper informed consent from the original studies, no further patient consent was required. The data usage adhered to ethical and legal norms, safeguarding patient rights and privacy.

## Conflicts of Interest

The authors declare no conflicts of interest.

## Funding

This work was supported by the Research‐Oriented Hospital Medical Research Project of the Affiliated Hospital of Jiangnan University (Grant YJY202308); the Natural Science Foundation of Jiangsu Province (Grant BK20240310); the Research Grant of Wuxi Municipal Health Commission (Grant Q202449); the Youth Talent Support Project of Wuxi Municipal (Grant TJXD‐2024‐216).

## Supporting Information

Additional supporting information can be found online in the Supporting Information section.

## Supporting information


**Supporting Information 1** Table S1: SNPs from 91 inflammatory cytokines as the instrumental variables.

**Supporting Information 2** Figure S1: Forest plots, funnel plots, scatter plots, and the Leave‐one‐out analyses of positive findings in Mendelian randomization analysis for inflammatory cytokines on monoclonal gammopathy of undetermined significance. (A) CXCL10 on monoclonal gammopathy of undetermined significance. (B) IL6 on monoclonal gammopathy of undetermined significance.

**Supporting Information 3** Figure S2: Forest plots, funnel plots, scatter plots, and the Leave‐one‐out analyses of the reverse Mendelian randomization analysis for monoclonal gammopathy of undetermined significance on (A) CXCL10 and (B) IL6.

**Supporting Information 4** Table S2: SNPs from 1400 metabolites as the instrumental variables.

**Supporting Information 5** Figure S3: Forest plots, funnel plots, scatter plots, and the Leave‐one‐out analyses of positive findings in Mendelian randomization analysis for metabolites on monoclonal gammopathy of undetermined significance. (A) Threonate level on monoclonal gammopathy of undetermined significance. (B) X‐22776 on monoclonal gammopathy of undetermined significance. (C) N‐acetylputrescine to (N(1) + N(8))‐acetylspermidine ratio on monoclonal gammopathy of undetermined significance. (D) Glucose to sucrose ratio on monoclonal gammopathy of undetermined significance.

**Supporting Information 6** Figure S4: Forest plots, funnel plots, scatter plots, and the Leave‐one‐out analyses of Mendelian randomization analysis for CXCL10 on (A) Threonate level, (B) X‐22776, (C) N‐acetylputrescine to (N(1) + N(8))‐acetylspermidine ratio and (D) Glucose to sucrose ratio.

## Data Availability

The data supporting the findings of this study are available in the EBI GWAS Catalog at https://www.ebi.ac.uk/gwas/. The accession numbers for 91 circulating cytokines range from GCST90274758 to GCST90274848. The accession number for monoclonal gammopathy of undetermined significance is GCST90043942. The accession numbers for 1091 blood metabolites and 309 metabolite ratios range from GCST90199621 to GCST90201020. You can also directly contact the corresponding author for further inquiries.
