# Supplementary figures and images for: Targeting the Inflammation–Metabolism Axis in MGUS: Causal Roles of CXCL10 Mediated by Blood Metabolites
Source: Mediators Inflamm. 2025 Dec 8;2025:8804923. doi: 10.1155/mi/8804923 (PMC12767486; doi:10.1155/mi/8804923)

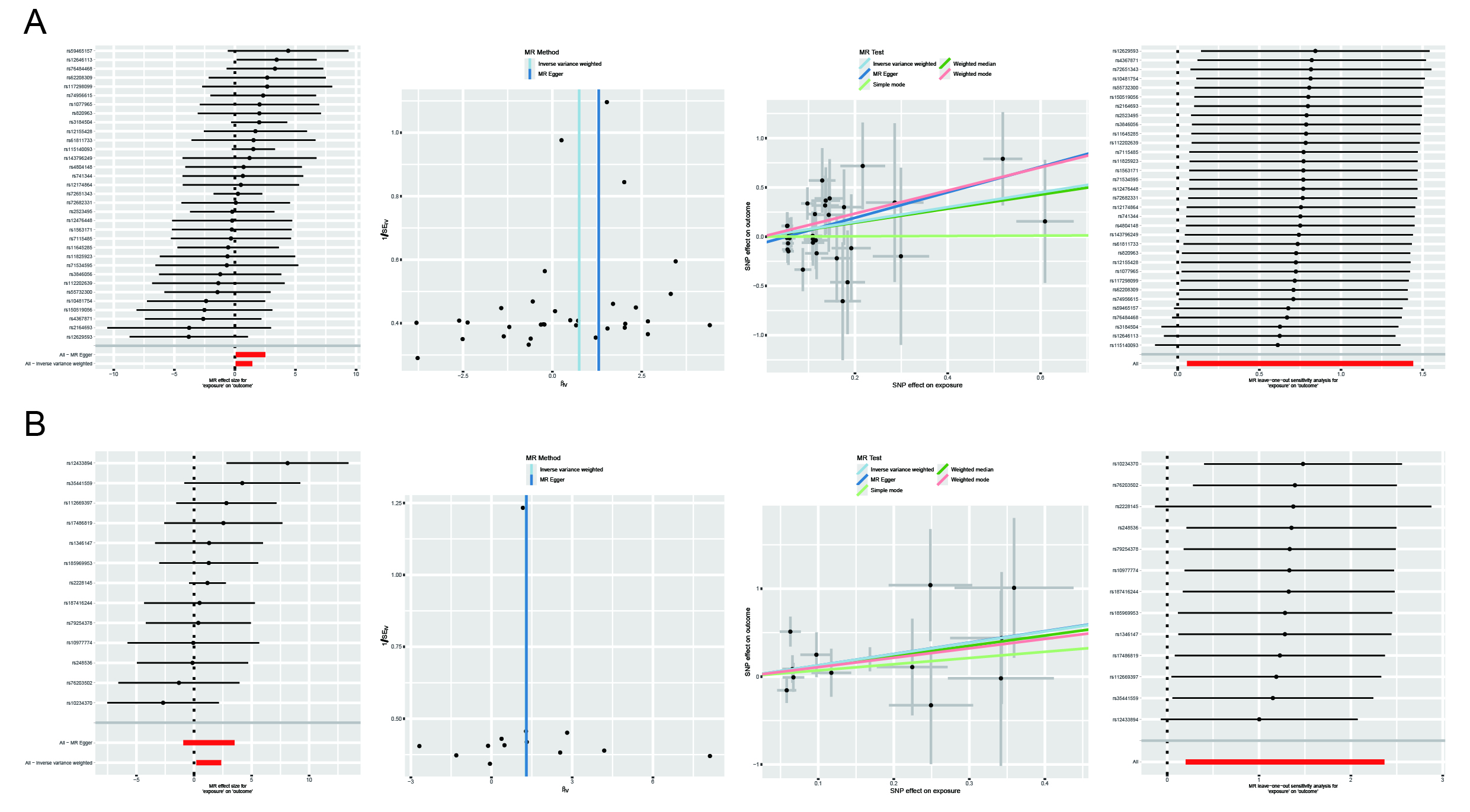

Supplement: Supplementary file 2 — Supporting Information 2 Figure S1: Forest plots, funnel plots, scatter plots, and the Leave‐one‐out analyses of positive findings in Mendelian randomization analysis for inflammatory cytokines on monoclonal gammopathy of undetermined significance. (A) CXCL10 on monoclonal gammopathy of undetermined significance. (B) IL6 on monoclonal gammopathy of undetermined significance. [file MI-2025-8804923-s006.jpg]

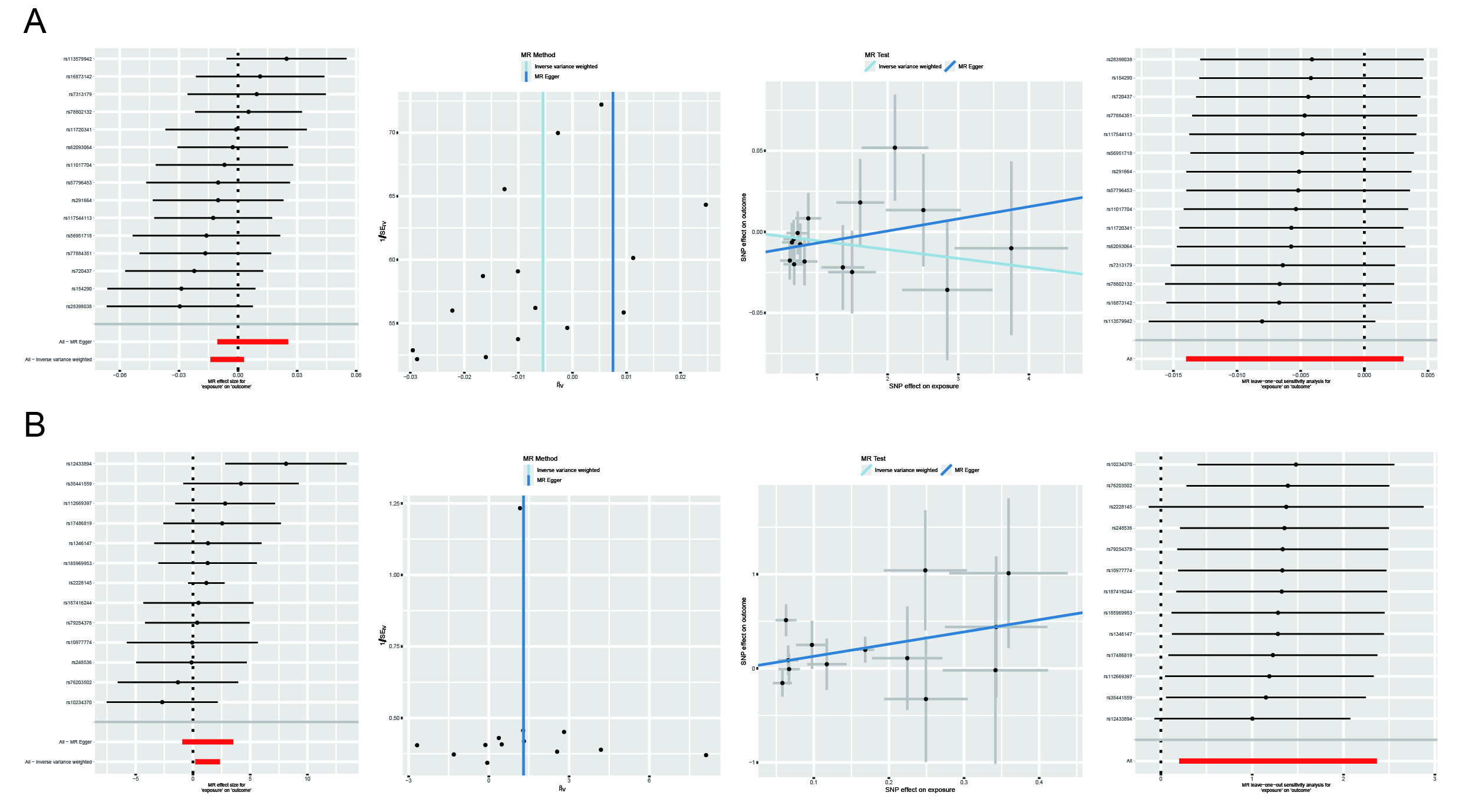

Supplement: Supplementary file 3 — Supporting Information 3 Figure S2: Forest plots, funnel plots, scatter plots, and the Leave‐one‐out analyses of the reverse Mendelian randomization analysis for monoclonal gammopathy of undetermined significance on (A) CXCL10 and (B) IL6. [file MI-2025-8804923-s004.jpg]

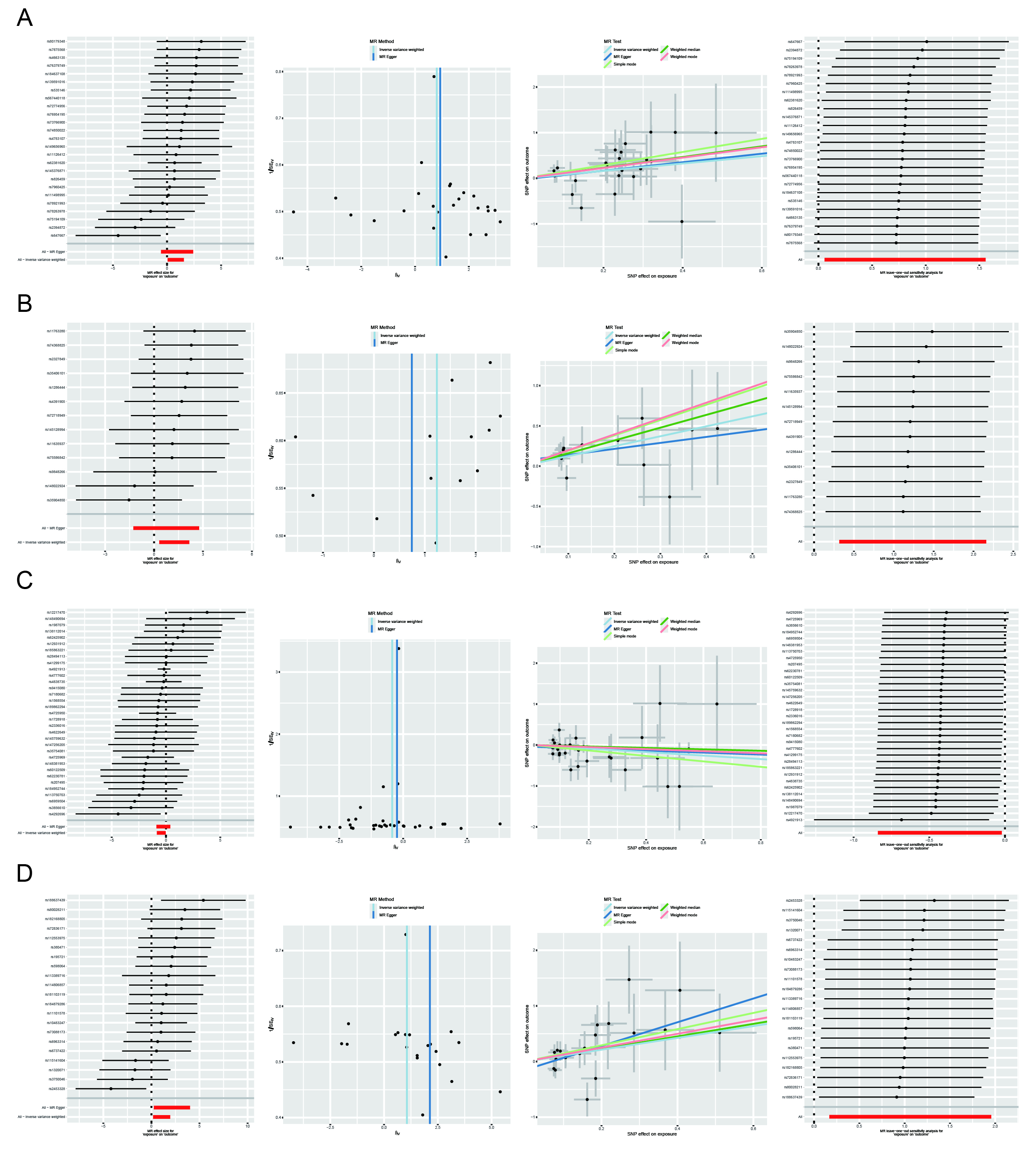

Supplement: Supplementary file 5 — Supporting Information 5 Figure S3: Forest plots, funnel plots, scatter plots, and the Leave‐one‐out analyses of positive findings in Mendelian randomization analysis for metabolites on monoclonal gammopathy of undetermined significance. (A) Threonate level on monoclonal gammopathy of undetermined significance. (B) X‐22776 on monoclonal gammopathy of undetermined significance. (C) N‐acetylputrescine to (N(1) + N(8))‐acetylspermidine ratio on monoclonal gammopathy of undetermined significance. (D) Glucose to sucrose ratio on monoclonal gammopathy of undetermined significance. [file MI-2025-8804923-s005.jpg]

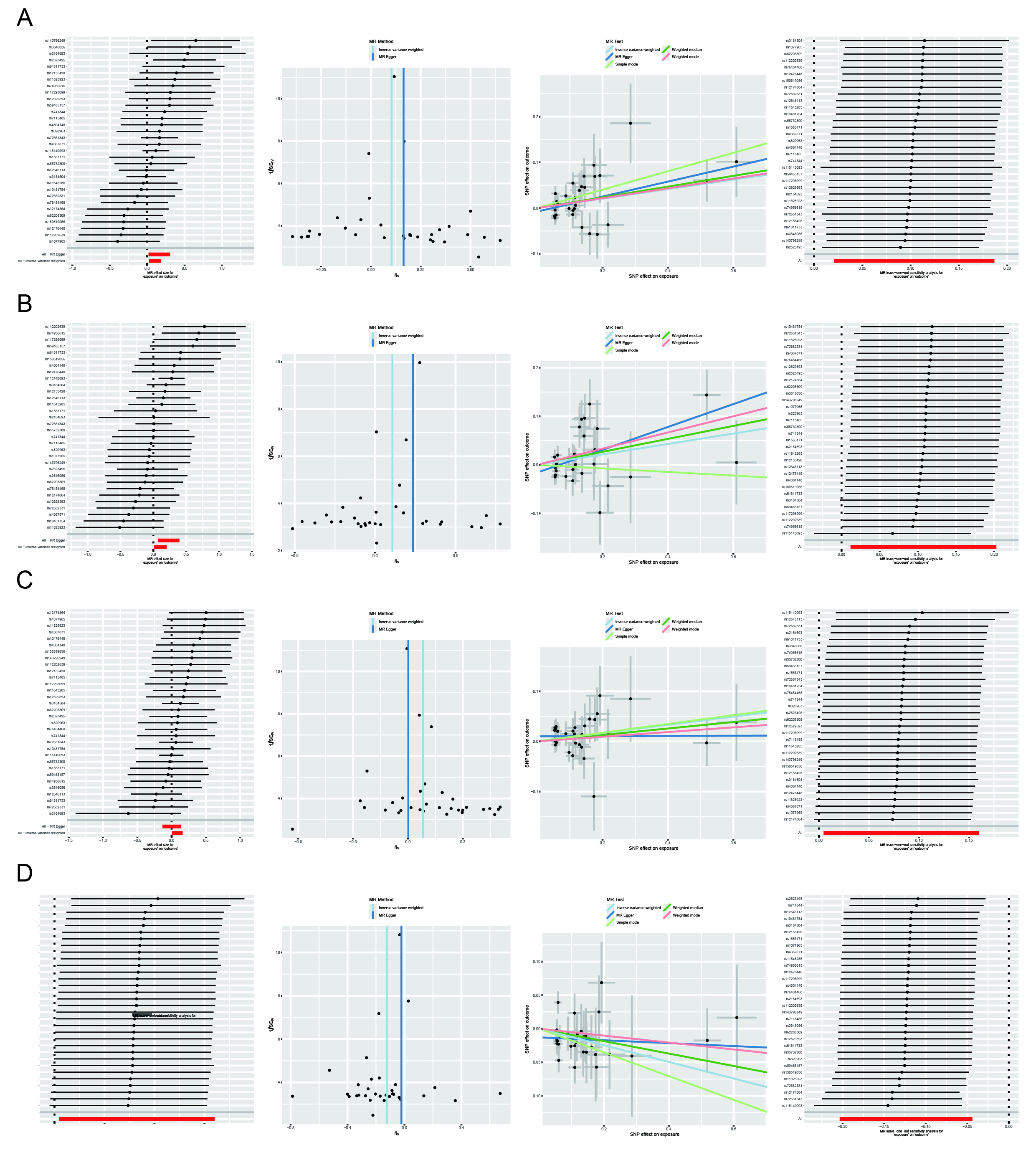

Supplement: Supplementary file 6 — Supporting Information 6 Figure S4: Forest plots, funnel plots, scatter plots, and the Leave‐one‐out analyses of Mendelian randomization analysis for CXCL10 on (A) Threonate level, (B) X‐22776, (C) N‐acetylputrescine to (N(1) + N(8))‐acetylspermidine ratio and (D) Glucose to sucrose ratio. [file MI-2025-8804923-s001.jpg]
